# Factors Involved in Preoperative Edema in High-Grade Gliomas

**DOI:** 10.7759/cureus.31379

**Published:** 2022-11-11

**Authors:** Takashi Saito, Masashi Mizumoto, Hsiang-Kuang Liang, Kei Nakai, Taisuke Sumiya, Takashi Iizumi, Hidehiro Kohzuki, Haruko Numajiri, Hirokazu Makishima, Takao Tsurubuchi, Masahide Matsuda, Eiichi Ishikawa, Hideyuki Sakurai

**Affiliations:** 1 Department of Radiation Oncology, University of Tsukuba Hospital, Tsukuba, JPN; 2 Division of Radiation Oncology, National Taiwan University Hospital, Taipei, TWN; 3 Department of Neurosurgery, University of Tsukuba Hospital, Tsukuba, JPN

**Keywords:** isocitrate dehydrogenase, magnetic resonance imaging, preoperative edema, radiotherapy, glioblastoma, glioma

## Abstract

Background

Expansion of preoperative edema (PE) is an independent poor prognostic factor in high-grade gliomas. Evaluation of PE provides important information that can be readily obtained from magnetic resonance imaging (MRI), but there are few reports on factors associated with PE. The goal of this study was to identify factors contributing to PE in Grade 3 (G3) and Grade 4 (G4) gliomas.

Methodology

PE was measured in 141 pathologically proven G3 and G4 gliomas, and factors with a potential relationship with PE were examined in univariate and multivariate analyses. The following eight explanatory variables were used: age, sex, Karnofsky performance status (KPS), location of the glioma, tumor diameter, pathological grade, isocitrate dehydrogenase (IDH)-1-R132H status, and Ki-67 index. Overall survival (OS) and progression-free survival (PFS) were calculated in groups divided by PE (<1 vs. ≥1 cm) and by factors with a significant correlation with PE in multivariate analysis.

Results

In univariate analysis, age (p = 0.013), KPS (p = 0.012), pathology grade (p = 0.004), and IDH1-R132H status (p = 0.0003) were significantly correlated with PE. In multivariate analysis, only IDH1-R132H status showed a significant correlation (p = 0.036), with a regression coefficient of -0.42. The median follow-up period in survivors was 38.9 months (range: 1.2-131.7 months). The one-, two-, and three-year OS rates for PE <1 vs. ≥1 cm were 77% vs. 68%, 67% vs. 44%, and 63% vs. 24% (p = 0.0001), respectively, and those for IDH1-R132H mutated vs. wild-type cases were 85% vs. 67%, 85% vs. 40%, and 81% vs. 21% (p < 0.0001), respectively. The one-, two-, and three-year PFS rates for PE <1 vs. ≥1 cm were 77% vs. 49%, 64% vs. 24%, and 50% vs. 18% (p = 0.0002), respectively, and those for IDH1-R132H mutated vs. wild-type cases were 85% vs. 48%, 77% vs. 23%, and 73% vs. 14% (p < 0.0001), respectively.

Conclusions

IDH1-R132H status was found to be a significant contributor to PE. Cases with PE <1 cm and those with the IDH1-R132H mutation clearly had a better prognosis.

## Introduction

Edema around a glioma is associated with malignancy of the tumor. Vascular endothelial growth factor (VEGF) secreted by malignant gliomas promotes the development of neovascular vessels, which results in increased permeability that causes edema around the tumor [1]. Such edema promotes tumor cell infiltration into the surrounding area, which extends the range over which the tumor can expand [2]. In Grade 3 (G3) and Grade 4 (G4) gliomas, extensive preoperative edema (PE) can be readily identified on magnetic resonance imaging (MRI) and has been found to be a poor prognostic factor [3-6]. The degree of edema can be classified into categories, but may also be described numerically [6,7]. This numerical approach permits the use of PE as an indicator that can be easily measured and is not limited by subjective differences among raters. The goal of this study is to identify factors associated with PE.

## Materials and methods

Patients and methods

Cases of high-grade gliomas with a pathology of G3 or G4 that received postoperative irradiation between 2011 and 2019 were extracted from our radiotherapy database. The pathology and pathological grade of glioma were determined according to the 2007 or 2016 World Health Organization (WHO) classification of tumors of the central nervous system (CNS) [8,9]. Of these cases, 141 were included in the study based on meeting the following criteria: (1) availability of preoperative, contrast-enhanced, T1-weighted MRI, T2-weighted MRI, or fluid-attenuated inversion recovery (FLAIR) MRI; and (2) a pathology report documenting the results of immunostaining for isocitrate dehydrogenase (IDH)-1-R132H status and Ki-67 index.

Treatment

All patients received radiotherapy after surgery. For treatment planning, computed tomography (CT) images were obtained at 2- to 3-mm intervals at the treatment position. Patients were immobilized in the supine position with a thermoplastic mask. The initial clinical target volume (CTV) included the entire surgical cavity and surrounding edema plus a 1.2-cm margin in G3 glioma and a 1.7-cm margin in G4 glioma. The second CTV was shrunk to the area including the entire surgical cavity and residual tumor plus a 1.2-cm margin in the G3 glioma and a 1.7-cm margin in the G4 glioma. The doses to the initial CTV and to the second CTV were 46-50 Gy in 23-25 fractions and 10-14 Gy in 5-7 fractions, respectively. The total irradiation dose was 60 Gy in 30 fractions in both the cases with G3 and G4 glioma. The planning target volume (PTV) was set as CTV plus 3 mm for setup error. Daily temozolomide (75 mg/m^2^) was used in patients with G4 glioma and G3 glioma as concurrent chemotherapy, except for anaplastic oligodendroglioma. In patients with anaplastic oligodendroglioma, two cycles of nimustine (80 mg/m^2^) were administered during radiotherapy.

Evaluation of preoperative edema

**Figure 1:**
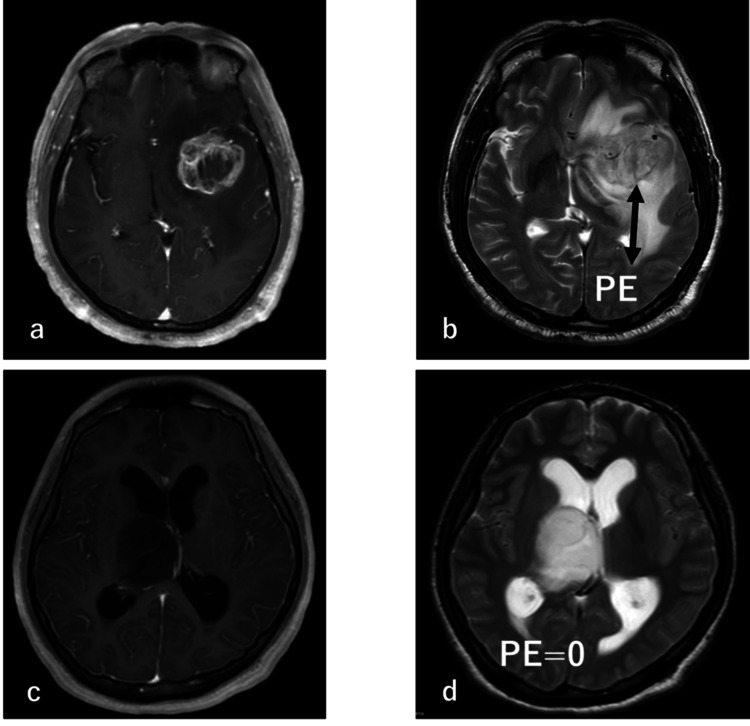
Definition of preoperative edema in cases with and without contrast enhancement. (a) Contrast-enhanced T1-weighted MRI in a case with contrast enhancement. (b) T2-weighted MRI in the case in (a). (c) Contrast-enhanced T1-weighted MRI in a case without contrast enhancement. (d) T2-weighted MRI in the case in (c). PE: preoperative edema; MRI: magnetic resonance imaging

The tumor was defined as the area enhanced on contrast-enhanced T1-weighted MRI, and PE was defined as the maximum length from the tumor edge to the high-signal area on T2-weighted or FLAIR MRI. To determine the maximum length, the slice showing the midline of the tumor was selected from axial, sagittal, and coronal sections, and the PE size was defined as the largest distance on these images (Figures 1a, 1b). A PE of 0 was assigned to cases in which the tumor showed no contrast and the boundary between the tumor and surrounding edema was unclear (Figures 1c, 1d). If the tumor was present at more than one site, the site for analysis was defined as that of the largest tumor. All images were issued with a reading report by at least one or more board-certified diagnostic radiologists and were reviewed by two radiation oncologists.

Statistical analysis

Data were analyzed using JMP version 13 (SAS institute, Cary, NC, USA). In univariate analysis, PE was the objective variable and eight clinical factors with a possible association with PE were used as explanatory variables, namely, age, sex (male and female), Karnofsky performance status (KPS), location of the glioma (frontal lobe and other locations), tumor diameter, pathological grade (G3 and G4), IDH1-R132H status (wild-type and mutated), and Ki-67 index. Categorical variables (sex, location, pathology grade, and IDH1-R132H status) were evaluated by the Mann-Whitney U test and numeric variables (age, KPS, tumor diameter, and Ki-67 index) were evaluated using Spearman correlation analysis.

Multivariate linear regression analysis was used to adjust for confounders and identify significant independent variables associated with PE using all eight of the clinical factors mentioned above as explanatory variables. The validity of the obtained multiple regression equation was confirmed by residual Q-Q plots that show the normality of residuals. Variance inflation factor (VIF) values were used to examine possible multi-collinearity among the explanatory variables.

Overall survival (OS) and progression-free survival (PFS) were calculated from the date of surgery using the Kaplan-Meier method. Differences in groups were compared by log-rank test. The cutoff value for PE was determined based on the receiver operating characteristic curve and was consequently set at 1 cm. Groups were also compared based on PE (<1 vs. ≥1 cm) and by factors that were significantly associated with PE in multiple regression analysis.

Ethical approval

This study was approved by the Ethical Review Committee and the University of Tsukuba Hospital Steering Committee (Tsukuba Clinical Research and Development Organization, H30-114). Written informed consent was obtained from patients before the administration of radiotherapy. The study was conducted in accordance with the principles outlined in the Declaration of Helsinki.

## Results

The clinical data for the 141 patients are shown in Table 1.

**Table 1:** Patient characteristics and tumor status.

Characteristics	Values (N = 141)
Age (years), median (range)	67 (14–88)
Gender, n	
Male	80
Female	61
Karnofsky performance status, n	
100	23
90	19
80	18
70	18
60	15
50	37
40	11
Surgical status, n	
Gross total resection	45
Subtotal resection	18
Partial resection	30
Biopsy	48
Pathology, n	
Glioblastoma	92
Anaplastic astrocytoma	25
Anaplastic oligodendroglioma	19
High-grade glioma	5
Gadolinium contrast pattern of tumors, n	
Enhanced	125
Not enhanced	16
Tumor location, n	
Frontal lobe	58
Temporal lobe	41
Parietal lobe	14
Occipital lobe	5
Basal ganglia	9
Cerebellum	6
Others	8
Tumor diameter (cm), median (range)	4.9 (1.0–10.2)
Isocitrate dehydrogenase 1-R132H status, n	
Wild-type	113
Mutated	28
Ki-67 index (%), median (range)	21 (1–90)
Preoperative edema (cm), median (range)	2.1 (0–8.4)

**Figure 2:**
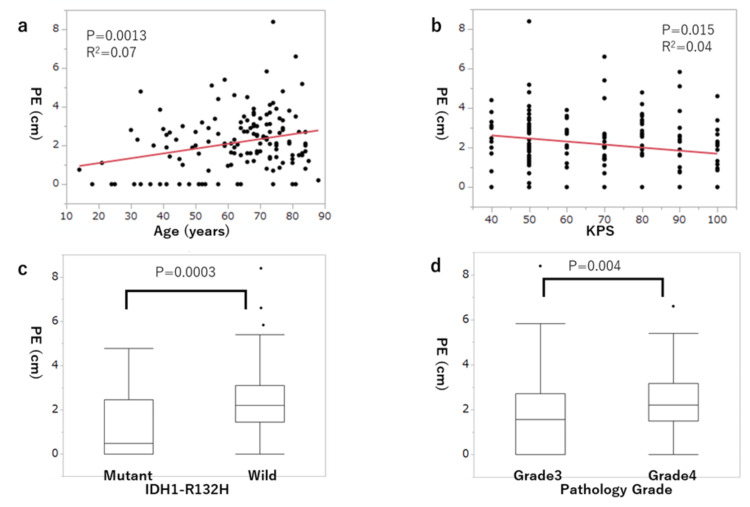
Relationships of preoperative edema with explanatory variables with a significant correlation in univariate analysis. (a) Age, (b) KPS, (c) IDH1-R132H status, and (d) pathology grade. PE: preoperative edema; KPS:  Karnofsky performance status; IDH: isocitrate dehydrogenase

In univariate analysis, age (p = 0.013), KPS (p = 0.012), IDH1-R132H status (p = 0.0003), and pathology grade (p = 0.004) were significantly correlated with PE (Figure 2). The other factors had no significant correlation. A normal distribution of residuals was shown in the Q-Q plot. VIF values showed no multi-collinearity among the eight explanatory variables. Of the four significant variables in univariate analysis, only IDH1-R132H status showed a significant correlation with PE in multivariate analysis (p = 0.036) (Table 2). The regression coefficient for IDH1-R132H status was -0.42. All p-values from univariate analysis and regression coefficients and p-values from multiple regression analysis are shown in Table 2.

**Table 2:** Results of univariate and multivariate analyses. IDH: isocitrate dehydrogenase

Variable	Univariate P-value	Regression coefficient	Multivariate P-value
Age (years)	0.013	0.014	0.12
Sex (male)	0.8	0.019	0.88
Karnofsky performance status	0.012	-0.0022	0.75
Location (frontal lobe)	0.24	-0.043	0.74
Tumor diameter (cm)	0.59	-0.0066	0.93
Pathology grade (G3)	0.004	-0.052	0.69
IDH1-R132H status (mutated)	0.0003	-0.42	0.036
Ki-67 index (%)	0.31	-0.0018	0.85

**Figure 3:**
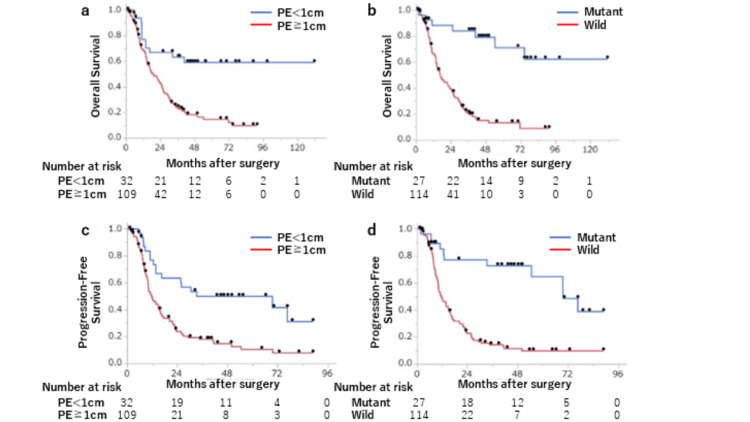
Kaplan-Meier curves for overall survival in cases with (a) PE <1 or ≥1 cm and (b) IDH1-R132H mutation or wild-type and for progression-free survival in cases with (c) PE <1 or ≥1 cm and (d) IDH1-R132H mutation or wild-type. PE: preoperative edema; IDH: isocitrate dehydrogenase

The median follow-up in survivors was 38.9 months (range = 1.2-131.7 months). OS rates based on PE and IDH1-R132H status are shown in Figure 3. The one-, two-, and three-year OS for PE <1 vs. ≥1cm were 77% vs. 68%, 67% vs. 44%, and 63% vs. 24% (p = 0.0001), respectively, and those for IDH1-R132H status (R132H mutation vs. wild-type) were 85% vs 67%, 85% vs 40%, and 81% vs 21% (p < 0.0001), respectively. The one-, two-, and three-year PFS rates for PE <1 vs. ≥1 cm were 77% vs. 49%, 64% vs. 24%, and 50% vs. 18% (p = 0.0002), respectively, and those for IDH1-R132H mutated vs. wild-type cases were 85% vs. 48%, 77% vs. 23%, and 73% vs. 14% (p < 0.0001), respectively.

## Discussion

The goal of this study was to identify factors contributing to PE in G3 and G4 gliomas. Multiple regression analysis showed that IDH1-R132H status significantly contributed to PE. Gliomas with IDH mutations are known to have minimally invasive features on MRI [10-12], with characteristics of low contrast enhancement, well-defined tumor borders, frontal lobe location, and a monogenic nature. However, there are few quantitative studies of peritumoral edema in gliomas with an IDH mutation. Dubinski et al. suggested that IDH wild-type glioblastomas have a high peritumoral edema volume/tumor volume (E/T) ratio [13]. The results of this study based on quantitative evaluation of edema are consistent with this finding. Liang et al. found that the E/T ratio contributes significantly to OS [7], and other reports have shown that PE (without consideration of tumor volume) is a prognostic factor in G3 or G4 glioma [3,6]. Therefore, the E/T ratio and PE are both useful prognostic factors for gliomas that can be objectively assessed on conventional MRI.

This study is significant in identifying factors contributing to PE and examining their influence using regression coefficients in multiple regression analysis. Short PE and IDH mutation were identified as favorable prognostic factors because cases with PE <1 cm and those with an IDH1-R132H mutation had clearly better OS. It is unclear why IDH mutations reduce peritumoral edema. Transforming growth factor-β and hypoxic signals are increased in IDH wild-type low-grade gliomas, and neovascularization in IDH-mutated and wild-type tumors has different molecular features [14]. Expression of VEGF, which is involved in neovascularization, is significantly lower in IDH-mutated gliomas [15]. These reports suggest that IDH wild-type gliomas have more aggressive neovascularization, which can lead to poorer outcomes. These findings are consistent with our results showing extended PE in IDH wild-type gliomas.

The WHO classification of tumors of CNS was revised in 2021 [16]. In the 2021 WHO classification of tumors of CNS, adult-type diffuse gliomas were classified into the following three categories based on IDH mutations: astrocytoma IDH-mutant, oligodendroglioma IDH-mutant and 1p/19q codeleted, and glioblastoma IDH-wild. In addition to the traditional morphological characteristics, the new classification placed more emphasis on molecular diagnosis. Because this study identified the presence of IDH mutations as a factor contributing to PE, PE may provide a clue from preoperative MRI to analogize the histology on the new classification.

The limitation of this study is that we only examined IDH1-R132H among IDH mutations. Although we performed immunostaining for IDH1-R132H, we did not search for other rare IDH variants. Therefore, it is possible that there is a mixture of variants other than IDH1-R132H among cases determined to be IDH wild-type in this study. However, IDH1-R132H is the most frequent mutation of IDH in primary tumors of the CNS, accounting for approximately 90% of such mutations [17]. Thus, even if a few rare IDH variants were mixed in with the IDH wild-type cases, the proportion would be small and unlikely to change the conclusions of the study.

## Conclusions

In an examination of PE in malignant gliomas using multiple regression analysis, IDH1-R132H status emerged as the only significant independent factor with a relationship with PE. Cases with PE <1 cm and those with an IDH1-R132H mutation had a clearly better prognosis.
